# Inactivation of Different *Salmonella enteriditis* Phage Types and Safety and Efficacy of Inactivated Products in Chicken

**DOI:** 10.1155/2021/8818308

**Published:** 2021-04-13

**Authors:** A. Akhtar, M. Hair-Bejo, Elawad A. Hussein, Zunita Zakaria

**Affiliations:** ^1^Department of Veterinary Pathology and Microbiology, Faculty of Veterinary Medicine, Universiti Putra Malaysia (UPM), 43400 Serdang, Selangor, Malaysia; ^2^Institute of Bioscience, Universiti Putra Malaysia (UPM), 43400 Serdang, Selangor, Malaysia

## Abstract

This study was conducted to inactivate *Salmonella enteriditis* phage types (SE pt) and to determine the safety and efficacy of inactivated SE pt in chickens. SE pt 1, 3A, 6A, 7, and 35 were inactivated and inoculated (0.20 mL) in 124 chickens divided into 6 groups (CV1, CV3A, CV6A, CV7, CV35, and CV0 as a control). Sampling was conducted on day 14 after inoculation (pi). Eight chickens from each group were separated on day 14 pi for oral challenge with 0.20 mL/chicken (10^10^ cfu/mL) SE pt 6A and designated CV1C, CV3AC, CV6AC, CV7C, CV35C, and CV0C as control chickens. On days 7 and 14 postchallenge (pc), 4 chickens from every group were sacrificed for sampling. There was no significant difference in the body weight between different groups. In challenged groups, there was no significant association between different tissues and isolation of *Salmonella* on days 7 and 14 pc. There was significance (*p* < 0.05) in isolation of *Salmonella* when CV0C group was compared with other challenged groups. Significance was not observed between different tissues with respect to induction of microscopic changes. Significance was not observed between day 7 pc and day 14 pc with respect to scoring of lesions induced. Clinical signs and gross lesions were also recorded. ELISA was applied. Only in CV3AC group, the mean antibody titer was 1359 on day 14 pc. The conclusion was that inactivated SE pt 3A and 6A were safe and efficacious for protection against *Salmonella enteriditis* infection in chickens.

## 1. Introduction

Human infections due to *Salmonella enterica* are considered as the major disease burdens worldwide. *Salmonella enterica* is a ubiquitous species, comprising more than 2600 different serovars. Theses serovars can be divided into typhoidal and nontyphoidal *Salmonella* (NTS) serovars [1]. However, poultry products, which serve as vehicles for *Salmonella,* play a major role in transmitting the microbe to human and animals to end up with huge economic losses. In European Union member states and United States of America, it was documented that the occurrence of chicken-borne *salmonellosis* ranged between 15.7% and 85.0% in humans [2].

Control measures on farms, to restrict the contamination of eggs with *Salmonella,* consist of strict farm biosecurity and routine chemical decontamination [3], while the methods to prevent gastrointestinal colonization in the host include the addition of organic acids to feed and water, use of probiotics and competitive exclusion products, and vaccination [3]. The application of vaccination, together with other control measures, is an important strategy to reduce *Salmonella* infection and thus to mitigate the food-borne risk of human disease [4].

Many types of vaccines have been developed for the prevention of *Salmonella* infection such as live attenuated vaccines which elicit both cell-mediated and humoral immune responses but still the problem exists [4]. Inactivated vaccines can provide the chicken with immunity that suppresses *Salmonella* colonization in organs and reduce shedding of the microbe in faeces [5]. Because inactivated vaccines constitute a well-known type of effective vaccines, inactivation of *Salmonella enteriditis* would considerably help in development of a vaccine for the control of Malaysian isolates of *Salmonella enteriditis*. The objectives of the study were to inactivate different *Salmonella enteriditis* phage types isolated in Malaysia and to determine the safety and efficacy of inactivated single *Salmonella enteriditis* phage types in SPF chickens.

## 2. Materials and Methods

All experimental procedures were undertaken in accordance with the Research Policy on Animal Ethics of Universiti Putra Malaysia.

### 2.1. Development of Inactivated *Salmonella enteriditis* Phage Types

*Salmonella enteriditis* phage types 1, 3A, 6A, 7, and 35 isolates were identified, propagated, and fermented in the bioreactor and then inactivated individually with formalin.

### 2.2. *Salmonella enteriditis* Isolates Phage Types

*Salmonella enteriditis* phage type 6A (UPM-0527), *Salmonella enteriditis* phage type 7 (UPM-0530), and *Salmonella enteriditis* phage type 35 (UPM-0525) were isolated from the liver samples of some commercial broiler chickens in Johor, Malaysia, whereas *Salmonella enteriditis* phage type 3A (UPM-0541) was isolated from faecal samples and *Salmonella enteriditis* phage type 1 (UPM-05) was isolated from the liver samples of commercial broilers in Melaka, Malaysia. All *Salmonella enteriditis* phage types were isolated in 2005 and then identified at the Laboratory of Enteric Pathogens, Centre of Infection, Colindate Avenue, London, United Kindom.

### 2.3. Identification of Candidate *Salmonella enteriditis* Phage Types

*Salmonella enteriditis* phage types 1, 3A, 6A, 7, and 35 were cultured on blood agar from stock and incubated for 24 hours at 37°C. From blood agar, a loop full of bacteria was cultured onto xylose lysine deoxycholate (XLD) and BGA and incubated for 24 hours at 37°C. Suspected *Salmonella* colonies were cultured onto triple sugar iron (TSI) and urea slants for biochemical tests. Suspected colonies were also used for Gram staining. *Salmonella* was confirmed by performing poly-O slide agglutination test.

### 2.4. Fermentation of Candidate *Salmonella enteriditis* Phage Types

The fermentation was performed individually for the 5 phage types of *Salmonella enteriditis*. The confirmed *Salmonella* colonies were cultured in 20 mL of Triptic Soya Broth (TSB) and incubated at 37°C for 24 hours. The culture was transferred to 4 litres (L) of (TSB) and incubated at 37°C for 18 hours. Then, it was ultimately transferred to cleaned and sterilized 50 L capacity bioreactor (Stirred Tank Reactor System S. No. 218/D50 Biostat D, B. Braun Biotech International) having 40 L of TSB for fermentation at 100 rpm, 37°C, and pH of 7. According to the standard procedure, the culture was fermented for 20 hours. The fermentation process was continuously monitored. The harvest was collected into two sterilized plastic drums. After fermentation, formaldehyde (0.7%) was added at once to inactivate the yield and mixed by manual shaking. The bioreactors were cleaned every time for the fermentation of next *Salmonella enteriditis* phage type.

### 2.5. Determination of Growth Curve

During fermentation, samples were collected in 20 mL sterilized glass bottles every 2 hours from the culture under fermentation. The growth rate was determined by making serial dilutions (10^−1^, 10^−2^, 10^−3^,…, 10^−10^) and plate count. Then 0.5 mL from culture under fermentation was transferred to one glass vial containing 4.50 mL of the diluent (TSB) to make 10^−1^. Next, 0.5 mL of diluted culture was transferred to a second glass vial containing 4.50 mL of the diluent (TSB) to make 10^−2^ dilution and so on; such technique was repeated 10 times. Thus, a serial dilution till 10^−10^ was carried out. For colony forming units/plate count, 0.1 mL of the last 3 dilutions (10^−8^, 10^−9^, and 10^−10^) was spread over XLD in two separate Petri dishes and incubated at 37°C for 24 hours. Two Petri dishes, without culture, were incubated as control.

### 2.6. Inactivation of *Salmonella*

Inactivation of the bacteria was done by the addition of formaldehyde 37% to the bacterial culture up to the final concentration of 0.7%. Formaldehyde 37% was added to the yield of each *Salmonella enteriditis* phage type after fermentation, mixed by manual shaking and stored at room temperature for 24 hours.

### 2.7. Determination of Percentage of Formalin for *Salmonella* Inactivation

The concentration of formalin for bacterial inactivation was determined for *Salmonella enteriditis* phage type 1 and *Salmonella enteriditis* phage type 6A before conducting the inactivation of different *Salmonella enteriditis* phage types. Different formalin concentrations;from 0.1%, 0.2%, 0.3%, etc…, 0.9% were tested for inactivation of the bacteria. The TSB culture containing 10^11^ bacterial culture (cfu)/mL (10^10^ cfu/0.1 mL) bacteria and 37% formaldehyde were used. The bacterial culture was prepared according to McFarland method by comparing the turbidity of bacterial culture with McFarland tube. The formalin was added drop by drop to the bacterial culture and mixed by gentle shaking. Eventually, formalin-added culture was incubated at 37°C. Furthermore, sterility was checked at 6, 12, 24, and 48 hours after incubation.

### 2.8. Sterility Test

The sterility test was performed to confirm the uniform and complete bacterial inactivation. Twenty mL from the formalin added harvest was collected in a sterile glass bottle. The full loop was streaked onto 2 XLD plates and kept for incubation at 37°C. The plates were examined for the bacterial growth after 24, 36, and 72 hours.

### 2.9. Adjuvant Preparation and Mixing

Aluminium potassium sulphate solution (10%) was prepared by dilution of 100 grams of aluminium potassium sulphate in one litre of distilled water. This adjuvant was added to the harvest in one drum and stored for 72 hours. It was added at a rate of one litre of the adjuvant to 10 litres of the harvest.

### 2.10. Filling and Labelling of Inactivated Products

The products were designated and labelled with the name of the respective *Salmonella enteriditis* phage types. V1, V3A, V6A, V7, and V35 represented formalin inactivated *Salmonella enteriditis* phage types 1, 3A, 6A, 7, and 35. Sterile plastic bottles were filled with formalin-inactivated bacteria, capped and stored at 4–8°C for further use in determination of the safety and efficacy of 5 different inactivated *Salmonella enteriditis* phage types, namely, V1, V3A, V6A, V7, and V35. The safety and efficacy of inactivated bacteria was determined in specific pathogen-free (SPF) chickens.

### 2.11. Determination of Safety and Efficacy of Inactivated Products

The formalin-inactivated *Salmonella enteriditis* phage types (V1, V3A, V6A, V7, and V35) were subcutaneously inoculated on the dorsal site of the neck of SPF chickens with a dosage of 0.20 mL of inactivated *Salmonella enteriditis* phage type. Other 10^10^ cfu/mL inocula for challenge were prepared with *Salmonella enteriditis* phage types 6A. For challenge, SPF chickens were orally inoculated with a dosage of 0.20 mL.

### 2.12. Experimental Design

One hundred twenty-four newly hatched SPF chickens were used. On arrival, 4 chickens were sacrificed to confirm the SPF status. Body weight, blood samples, and faecal swabs were collected. Samples for the isolation of *Salmonella* and histopathology were collected. The remaining chickens were divided into 6 groups of 20 chickens. The groups were CV1, CV3A, CV6A, CV7, CV35, and CV0 representing the chickens inoculated with inactivated *Salmonella enteriditis* phage types 1, 3A, 6A, 7, and 35 and one group of uninoculated chickens as a control, respectively.

The chickens were subcutaneously inoculated with inactivated *Salmonella enteriditis* phage types on the dorsal site of the neck at a dose of 0.20 mL/chicken (10^10^ cfu/mL). Each group was kept in a separate cage. Chickens were provided with antibiotic-free feed and water. Sampling was conducted on day 14 postinoculation (pi), prior to the challenge of the chickens with *Salmonella enteriditis* phage type 6A (UPM-0527). Four chickens were taken from each of the six groups and weighed. Before sacrifice, cloacal swabs and blood samples were collected. Examination of gross lesions was performed, and samples were collected from all chickens for the isolation of *Salmonella* and histopathology. The serum samples were separated and stored at −20 C for further application of ELISA.

Out of the remaining 16 chickens in each group, 8 chickens from each group were separated in a new room on day 14 pi for oral challenge with 0.20 mL/chicken (10^10^ cfu/mL) *Salmonella enteriditis* phage type 6A (UPM-0527) and designated as CV1C, CV3AC, CV6AC, CV7C, CV35C, and CV0C. In other words, the groups CV1C, CV3AC, CV6AC, CV7C, and CV35C represented the chickens inoculated with inactivated products (V1, V3A, V6A, V7, and V35), respectively, and challenged with *Salmonella enteriditis* phage type 6A, whereas CV0C represented the control chickens which were not inoculated but challenged with *Salmonella enteriditis* phage type 6A. After challenge, the chickens in each group—CV1C, CV3AC, CV6AC, CV7C, and CV35C—were kept in separate cages and provided with antibiotic-free feed and water. On days 7 and 14 postchallenge (pc), 4 chickens from groups CV1, CV3A, CV6A, CV7, CV35, and CV0 and also groups CV1C, CV3AC, CV6AC, CV7C, CV35C, and CV0C were sacrificed for sampling. All chickens were weighed and sacrificed after cloacal swabs, and blood samples were collected. Samples for the isolation of *Salmonella* and histopathology were collected from all chickens (Table 1).

### 2.13. Isolation of *Salmonella*

Samples of midintestinal content, caecal contents, cloacal swab, blood, liver, and spleen were collected from chickens for isolation and identification of *Salmonella* [6].

### 2.14. Histopathology

Samples of ileum, caecum, bursa of Fabricius, liver, and spleen were collected and fixed in 10% buffered formalin. The tissues were processed according to the standard technique of HE staining [7].

### 2.15. Enzyme-Linked Immunosorbent Assay (ELISA)

ELISA technique was applied on the serum collected according to the protocol recommended by Biocheck Poultry Immunoassays SE Antibody Test Kit, UK.

### 2.16. Statistical Analysis

Using IBM SPSS Statistics 25 software, the data was analyzed with one-way analysis of variance (ANOVA) and Tukey's honestly significant difference (HSD) pairwise multiple comparison procedure to determine the significance on body weight gain [8]. Pearson's chi-square test was used to analyse the data of bacterial isolation in different tissues. One-way ANOVA with repeated measures was applied to analyse the data of bacterial isolation among different groups and also for the analysis of scoring of microscopic lesions. However, for comparison between days with respect to the severity of lesions in tissues, *t-*test technique was applied.

## 3. Results

### 3.1. Growth Curve

The bacterial growth was counted every 2 hours during fermentation. It was observed that the bacteria were multiplying well throughout the fermentation. There were variations in the growth rates of different *Salmonella enteriditis* phage types at 2, 4, 6, 8, 10, 12, 14, 16, 18, and 20 hours after fermentation.

### 3.2. Percentage of Formalin for Inactivation of Bacteria

The formalin concentrations at the level 0.5% and above were able to completely inactivate the bacteria. No growth was observed in the cultures at 6, 12, 24, 36, and 48 hours. However, when the concentrations of formalin were 0.3 and 0.4%, the bacterial growths were noticed in the cultures.

### 3.3. Clinical Signs and Mortality Cases after Inoculation with Inactivated Products and Challenge with *Salmonella enteriditis* Phage Type 6A

Groups of controls: in the group CV0, neither abnormal clinical signs nor mortality cases were observed throughout the experiment. In the group CV0C, depression, anorexia, ruffled feathers, and diarrhoea were observed from day 2 postchallenge (pc) till the end of the experiment. One chicken died on day 3 pc.

Groups without challenge and groups with challenge: in the groups CV1, CV3A, CV6A, CV7, and CV35, no abnormal clinical signs and mortality cases were observed throughout the experiment.

In CV1C group, the chickens were depressed on days 1 and 2 pc. Mortality cases were not observed. In the group CV3AC, the chickens were depressed at hour 12 pc. Then, chickens recovered at hour 24 pc. Then, no abnormal sign was observed till the end of the experiment. In the group CV6AC, the chickens did not show abnormal clinical signs. However, only one chicken was depressed and standing aside at hour 6 pc. Mortality cases were not observed. In the group CV7C, the chickens showed depression and anorexia on days 1 and 2 pc. Ruffled feathers were observed on day 3 pc. In the group CV35C, depression and anorexia were observed on day 1 pc. Ruffled feathers were observed on day 3 pc till the end of the experiment. Mortality cases were not reported.

### 3.4. Body Weight

Body weight of controls: in the group CV0, the body weight increased continuously throughout the experiment. It was 35.00 ± 2.10 g and 124.00 ± 4.80 g on days 0 and 14 pi, respectively, and 201.40 ± 4.60 g and 325.00 ± 5.70 g on days 7 and 14 pc, respectively. There was no significant difference when it was compared to CV0C group at days 7 and 14 pc. In the group CV0C, the body weight was 195.00 ± 3.60 and 329.00 ± 2.70 g on days 7 and 14 pc, respectively.

Body weight in chickens without challenge and chickens with challenge: in the group CV1, the body weight was 35.00 ± 2.10 g and 317.40 ± 5.00 g on days 0 and 14 pi, respectively, and 205.00 ± 5.20 g and 327.50 ± 4.00 g on days 7 and 14 pc, respectively. There was no significant difference when it was compared to CV1C group on days 7 and 14 pc. In the group CV1C, the body weight was 202.00 ± 3.90 g and 317.40 ± 5.00 g on days 7 and 14 pc, respectively.

In the group CV3A, the body weight was 35.00 ± 2.10 g and 122.50 ± 1.80 g on days 0 and 14 pi, respectively, and 201.30 ± 2.30 g and 324.30 ± 3.40 g on days 7 and 14 pc, respectively. There was no significant difference when it was compared to CV3AC group. In the group CV3AC, the body weight was 203.80 ± 2.50 g and 319.30 ± 6.70 g on days 7 and 14 pc, respectively.

In the group CV6A, the body weight was 35.00 ± 2.10 g and 127.00 ± 2.50 g on days 0 and 14 pi, respectively, and 199.60 ± 3.40 g and 324.00 ± 2.40 g on days 7 and 14 pc, respectively. There was no significant difference when it was compared to CV6AC. In the group CV6AC, the body weight was 201.00 ± 2.10 g and 320.00 ± 9.10 g on days 7 and 14 pc, respectively.

In the group CV7, the body weight was 35.00 ± 2.10 g and 127.50 ± 4.40 g on days 0 and 14 pi, respectively, and 207.00 ± 3.00 g and 325.30 ± 6.80 g on days 7 and 14 pc, respectively. There was no significant difference when it was compared to CV7C. In the group CV7C, the body weight was 200.40 ± 8.10 g and 324.30 ± 7.90 g on days 7 and 14 pc, respectively.

In the group CV35, the body weight was 35.00 ± 2.10 g and 124.50 ± 2.70 g on days 0 and 14 pi, respectively, and 206.30 ± 3.50 g and 322.50 ± 7.20 g on days 7 and 14 pc, respectively. There was no significant difference when it was compared to CV35C. In the group CV35C, the body weight was 199.00 ± 6.50 g and 304.03 ± 7.90 g on days 7 and 14 pc, respectively.

### 3.5. Isolation of *Salmonella*

Isolation of *Salmonella* from controls: in the group CV0, *Salmonella* was not isolated from all samples collected throughout the experiment. *Salmonella* was not isolated from all chickens prior to inoculation of *Salmonella enteriditis* (day 0).

**Figure 1:**
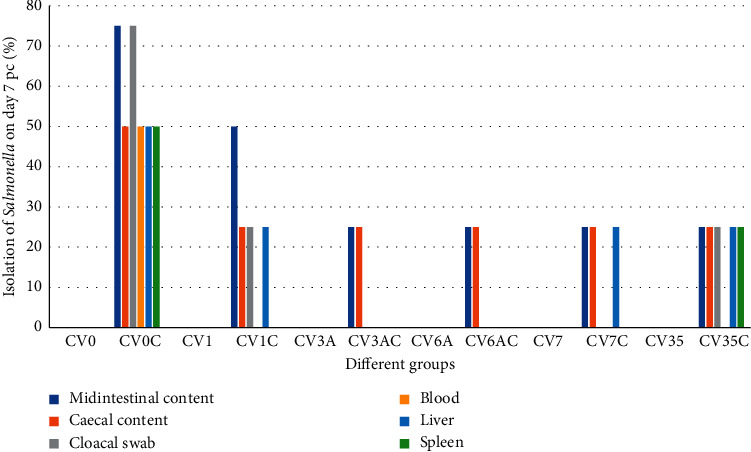
Isolation of *Salmonella* from different tissues of SPF chickens on day 7 pc. Percentage of isolation in all tissues is either 25%, 50%, or 75%.

**Figure 2:**
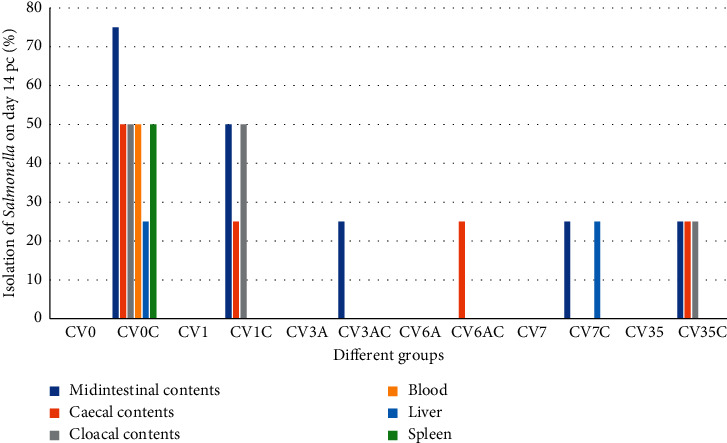
Isolation of *Salmonella* from different tissues of SPF chickens on day 14 pc. Percentage of isolation in all tissues is either 25%, 50%, or 75%.

In the group CV0C, *Salmonella* was isolated from all samples but with different percentages. These percentages were either 25%, 50%, or 75% (Figures 1-2).

Isolation of *Salmonella* from chickens inoculated with inactivated *Salmonella enteriditis* phage type 1 without challenge and chickens with challenge: In the group CV1, *Salmonella* was not isolated from all samples collected throughout the experiment. In the group CV1C, it was isolated from different samples with some variations (either 25% or 50%). However, it was also not isolated from blood and spleen on days 7 and 14 pc and from liver on day 14 pc (Figures 1-2).

Isolation of *Salmonella* from chickens inoculated with inactivated *Salmonella enteriditis* phage type 3A without challenge and chickens with challenge: in the group CV3A, *Salmonella* was not isolated from all samples collected throughout the experiment. In the group CV3AC, *Salmonella* was isolated with a percentage of 25% from midintestinal contents on days 7 and 14 pc and from caecal contents on day 14 pc. In other tissues, it was not isolated throughout the experiment (Figures 1-2).

Isolation of *Salmonella* from chickens inoculated with inactivated *Salmonella enteriditis* phage type 6A without challenge and chickens with challenge: in the group CV6A, *Salmonella* was not isolated from all samples collected throughout the experiment. In the group CV6AC, it was isolated with a percentage of 25% from midintestinal contents on day 7 pc and from caecal contents on days 7 and 14 pc. In other tissues, it was not isolated throughout the experiment (Figures 1-2).

Isolation of *Salmonella* from chickens inoculated with inactivated *Salmonella enteriditis* phage type 7 without challenge and chickens with challenge: in the group CV7, *Salmonella* was not isolated from all samples collected throughout the experiment. In the group CV7C, the isolation percentage was 25% from midintestinal contents and liver on days 7 and 14 pc and from caecal contents on day 7 pc. However, in other tissues, it was not isolated throughout the experiment (Figures 1-2).

Isolation of *Salmonella* from chickens inoculated with inactivated *Salmonella enteriditis* phage type 35 without challenge and chickens with challenge: in the group CV35, *Salmonella* was not isolated from all samples collected throughout the experiment. In the group CV35C, it was isolated with a percentage of 25% from midintestinal, caecal contents on days 7 and 14 pc and cloacal swab, liver, and spleen on day 7 pc, whereas it was not isolated from other tissues (Figures 1-2).

### 3.6. Statistical Analysis for Isolation of *Salmonella* from Different Tissues of Chickens Inoculated with Inactivated Products and Challenge

Chi-square association technique was applied to determine if there was an association between a certain tissue and the isolation of *Salmonella* on days 7 and 14 pc. It was found that there was no significant association between different tissues and the isolation of *Salmonella* on days 7 and 14 pc (Tables 2 and 3).

### 3.7. Statistical Analysis for Isolation of *Salmonella* from Different Groups of Chickens Inoculated with Inactivated Products and Challenge on Day 7 pc

The value of isolation of *Salmonella* in group CV0C on day 7 pc was calculated by the addition of the value of isolation in midintestinal content, the value of isolation in cloacal swap, the value in blood, and the value in liver and the value in spleen. Then, the mean ± standard deviation and standard error were obtained. The same technique was applied for groups CV1C, CV3AC, CV6AC, CV7C, and CV35C by application of one-way repeated measures ANOVA (Tables 4 and 5). Eventually, the differences of means for isolation of *Salmonella* between the individual groups were obtained. The significance (*p* < 0.05) was observed only in case of difference between CV0C (control group) and any other group (Table 6).

### 3.8. Statistical Analysis for Isolation of *Salmonella* from Different Groups of Chickens Inoculated with Inactivated Products and Challenge on Day 14 pc

The overall value for isolation of *Salmonella* in group CV0C on day 14 pc was calculated by the addition of the value of isolation in midintestinal content, the value of isolation in cloacal swap, the value in blood, the value in liver, and the value in spleen. Then, the mean ± standard deviation and standard error were obtained. The same technique was applied for groups CV1C, CV3AC, CV6AC, CV7C, and CV35C by the application of one-way repeated measures ANOVA (Tables 7 and 8). Eventually, the differences of means for isolation of *Salmonella* between the individual groups were obtained. The significance (*p* < 0.05) was observed only in case of difference between CV0C (control group) and any other group (Table 9).

### 3.9. Gross Lesion

Gross lesions in controls: in group CV0, gross lesions were not observed throughout the experiment.

In the group CV0C, gross lesions were not observed apart from enlargement of bursa of Fabricius in one chicken on day 14 pc and enlargement of bursa of Fabricius and liver in one chicken died on day 3 pc.

Gross lesions in chickens inoculated with different inactivated products without challenge and chickens with different inactivated products and challenge: in group CV1 and group CV1C, gross lesions were not observed throughout the experiment. In group CV3A and group CV3AC, gross lesions were not observed throughout the experiment. In group CV6A and group CV6AC, gross lesions were not observed throughout the experiment. In group CV7 and group CV7C, gross lesions were not observed throughout the experiment. In group CV35 and group CV35C, gross lesions were not observed throughout the experiment.

### 3.10. Microscopic Lesions in Ileum

**Figure 3:**
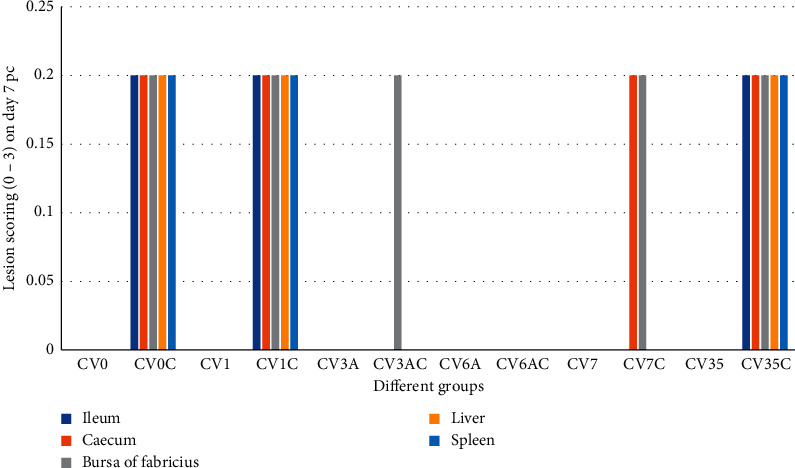
Lesion scoring of ileum, caecum, bursa of Fabricius, liver, and spleen tissues of SPF chickens in different groups on day 7 pc.

**Figure 4:**
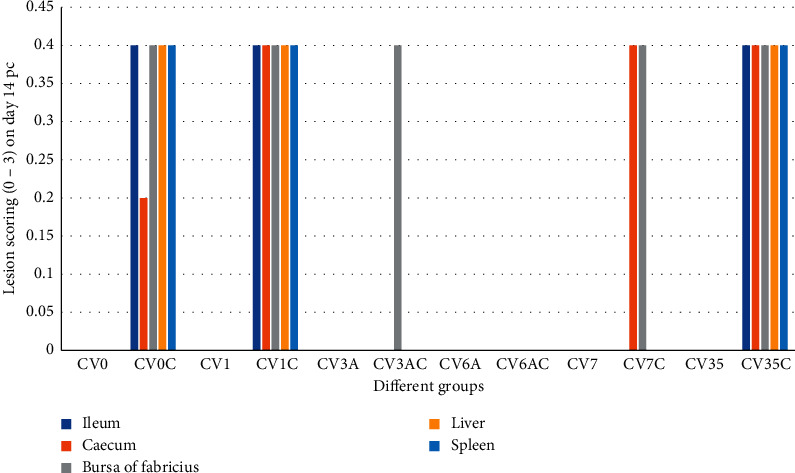
Lesion scoring of ileum, caecum, bursa of Fabricius, liver, and spleen tissues of SPF chickens in different groups on day 14 pc.

Microscopic lesions in ileum of controls: in the group CV0, the microscopic lesions were not detected throughout the experiment (scoring of 00 ± 00) (Figures 3-4). In the group CV0C, mild heterophilic infiltration and congestion (scoring of 0.20 ± 0.20) were detected on day 7 pc, and mild-to-moderate heterophilic infiltration and congestion (scoring of 0.40 ± 0.20) were detected on day 14 pc (Figures 3-4).

Microscopic lesions in ileum of chickens inoculated with different inactivated products without challenge and ileum of chickens inoculated with different inactivated products and challenge: in the group CV1, the lesions were not detected throughout the experiment (scoring of 00 ± 00). In group CV1C, mild-to-moderate heterophilic infiltration and congestion were detected on day 7 and 14 pc. Scoring of 0.20 ± 0.20 and 0.40 ± 0.20 were recorded on days 7 and 14 pc, respectively (Figures 3-4).

In group CV3A, group CV3AC, group CV6A, group CV6AC, group CV7, group CV7C, and group CV35, the lesions were not detected throughout the experiment. Scoring of 0.00 ± 0.00 was recorded. However, in group CV35C, mild heterophilic infiltration and congestion were detected on day 7 and 14 pc. Scoring of 0.20 ± 0.20 and 0.40 ± 0.20 were recorded on days 7 and 14 pc, respectively (Figures 3-4).

### 3.11. Microscopic Lesions in Caecum

Microscopic lesions in caecum of controls: in the group CV0, no microscopic lesions were detected throughout the experiment (scoring of 00 ± 00). In the group CV0C, mild heterophilic infiltration and congestion (scoring of 0.20 ± 0.20) were detected on days 7 and 14 pc (Figures 3-4).

Microscopic lesions in caecum of chickens inoculated with different inactivated products without challenge and chickens with different inactivated products and challenge: in group CV1, group CV3A, group CV3AC, group CV6A, group CV6AC, group CV7, and group CV35, lesions were not detected throughout the experiment. Scoring of 0.00 ± 0.00 was recorded. However, in group CV1C, group CV7C, and group CV35C, mild heterophilic infiltration and congestion were detected on day 7 and 14 pc. Scoring of 0.20 ± 0.20 and 0.40 ± 0.20 were recorded on days 7 and 14 pc, respectively (Figures 3-4).

### 3.12. Microscopic Lesions in Bursa of Fabricius

Microscopic lesions in bursa of Fabricius of controls: in the group CV0, no microscopic lesions were detected throughout the experiment (scoring of 00 ± 00). In the group CV0C, mild-to-moderate heterophilic infiltration, congestion, and necrosis were detected on days 7 and 14 pc. Scoring of 0.20 ± 0.20 and 0.40 ± 0.20 were recorded on days 7 and 14 pc, respectively (Figures 3-4).

Microscopic lesions in bursa of Fabricius of chickens inoculated with different inactivated products without challenge and chickens with different inactivated products and challenge: in group CV1, group CV3A, group CV6A, group CV6AC, group CV7, and group CV35, lesions were not detected throughout the experiment. Scoring of 0.00 ± 0.00 was recorded, while in group CV1C, group CV3AC, group CV7C, and group CV35C, mild-to-moderate heterophilic infiltration, congestion, and necrosis were detected on days 7 and 14 pc. Scoring of 0.20 ± 0.20 and 0.40 ± 0.20 were recorded on days 7 and 14 pc, respectively. However, necrosis was absent in CV7C (Figures 3-4).

### 3.13. Microscopic Lesions in Liver

Microscopic lesions in liver of controls: in the group CV0, microscopic lesions were not detected throughout the experiment (scoring of 00 ± 00). In the group CV0C, mild heterophilic infiltration and congestion were detected on day 7 pc. Moderate heterophilic infiltration and congestion were detected on day 14 pc. Scoring of 0.20 ± 0.20 and 0.40 ± 0.20 were recorded on days 7 and 14 pc, respectively (Figures 3-4).

Microscopic lesions in liver of chickens inoculated with different inactivated products without challenge and chickens with different inactivated products and challenge: in group CV1, group CV3A, group CV3AC, group CV6A, group CV6AC, group CV7, group CV7C, and group CV35, lesions were not detected throughout the experiment. Scoring of 0.00 ± 0.00 was recorded, whereas in group CV1C and group CV35C, mild-to-moderate heterophilic infiltration and congestion were detected on days 7 and 14 pc. Scoring of 0.20 ± 0.20 and 0.40 ± 0.20 were recorded on days 7 and 14 pc, respectively (Figures 3-4).

### 3.14. Microscopic Lesions in Spleen

Microscopic lesions in spleen of controls: in the group CV0, microscopic lesions were not detected throughout the experiment (scoring of 00 ± 00). In the group CV0C, mild heterophilic infiltration and congestion were detected on day 7 pc. Mild-to-moderate heterophilic infiltration and congestion were detected on day 14 pc. Scoring of 0.20 ± 0.20 and 0.40 ± 0.20 were recorded on days 7 and 14 pc, respectively (Figures 3-4).

Microscopic lesions in spleen of chickens inoculated with different inactivated products without challenge and chickens with different inactivated products and challenge: in group CV1, group CV3A, group CV3AC, group CV6A, group CV6AC, group CV7, group CV7C, and group CV35, lesions were not detected throughout the experiment. Scoring of 0.00 ± 0.00 was recorded, whereas in group CV1C and group CV35C, mild-to-moderate heterophilic infiltration and congestion were detected on days 7 and 14 pc. Scoring of 0.20 ± 0.20 and 0.40 ± 0.20 were recorded on days 7 and 14 pc, respectively (Figures 3-4).

### 3.15. Statistical Analysis for Scoring of Lesions Induced by *Salmonella* in Different Tissues of Chickens Inoculated with Inactivated Products and Challenge on Day 7 pc

Heterophilic infiltration (mild to moderate) and congestion were detected with different scorings in almost all tissues on day 7 pc. The scoring of this lesion in the ileum in group CV0C was added to the scoring of the same lesion in ileum in group CV1C, the scoring of the same lesion in ileum of group CV3AC, the scoring of the same lesion in ileum in CV6AC, the scoring of the same lesion in CV7C, and the scoring of the same lesion in CV35C. One-way ANOVA with repeated measures was applied. The mean, standard deviation, and standard error were calculated. The same technique was applied for this lesion in the caecum, bursa of Fabricius, liver, and spleen. It was found that there were no significant differences between the means of different tissues when compared with each other (Tables 10–12). However, necrosis was not implicated because it was only detected in the bursa of Fabrius.

### 3.16. Statistical Analysis for Scoring of Lesions Induced by *Salmonella* in Different Tissues of Chickens Inoculated with Inactivated Products and Challenge on Day 14 pc

Heterophilic infiltration (mild to moderate) and congestion were detected with different scorings in almost all tissues on day 14 pc. The scoring of this lesion in ileum in the group CV0C was added to the scoring of the same lesion in ileum in group CV1C, added to the scoring of the same lesion in ileum of group CV3AC, added to the scoring of the same lesion in ileum in CV6AC, added to the scoring of the same lesion in CV7C, and added to the scoring of the same lesion in CV35C. One-way ANOVA with repeated measures was applied. The mean, standard deviation, and standard error were calculated. The same technique was applied for this lesion in the caecum, bursa of Fabricius, liver, and spleen. It was found that there were no significant differences between the means of different tissues when they were compared with each other (Tables 13–15).

### 3.17. Comparison of Day 7 and Day 14 pc with Respect to Severity of Lesions Induced by *Salmonella* in Different Tissues of Chickens Inoculated with Inactivated Products and Challenge

The summation of overall scoring of heterophilic infiltration and congestion, in different tissues (ileum, caecum, bursa of Fabricius, liver, and spleen), on day 7 pc was calculated for group CV0C. Then, the summation of overall scoring for the same lesion was calculated for groups CV1C, CV3AC, CV6AC, CV7C, and CV35C. Next, the scoring of this lesion was obtained for all groups together on day 7 pc.

This technique was applied for the same lesion in all groups on day 14 pc. Eventually, the means, standard deviations, and standard errors were obtained for the scoring of the lesion on day 7 and day 14 pc and compared by the application of paired-samples *t*-test. However, the means of scoring of lesions on day 7 pc and day 14 pc were not significantlydifferent (Tables 16 and 17).

### 3.18. ELISA

Application of ELISA for detection of *Salmonella enteriditis* in controls: in group of CV0 and group of CV0C, *Salmonella enteriditis* antibodies were not determined throughout the experiment (Table 18).

Application of ELISA for detection of *Salmonella enteriditis* in chickens inoculated with different inactivated products without challenge and chickens with different inactivated products and challenge: in group CV1, group CV1C, group CV3A, group CV6A, group CV6AC, group CV7, group CV7C, group CV35, and group CV35C, *Salmonella enteriditis* antibodies were not determined throughout the experiment (Table 18). But in group CV3AC, the mean antibody titer was 1359 on day 14 pc (Table 18).

## 4. Discussion

The study showed that different inactivated *Salmonella enteriditis* phage types could provide different levels of prevention; in other words, the inactivated products had potential to mitigate the experimental *Salmonella enteriditis* phage type 6A infection in SPF chickens. It was observed that different inactivated *Salmonella enteriditis* phage types could reduce the load of *Salmonella enteriditis* in the host, but could not eliminate the organism [9]. The overall results of the study indicated that both inactivated *Salmonella enteriditis* phage types 3A and 6A were safe and effective and might contribute to the alleviation of *Salmonella enteriditis* phage type 6A infection in poultry.

The inoculation of different inactivated *Salmonella enteriditis* phage types alleviated the clinical picture of the challenge when compared with the control. All groups inoculated with challenge (CV1C, CV3AC, CV6AC, CV7C, and CV35C) underwent milder clinical signs than uninoculated and challenged control group (CV0C). The variation, in alleviated clinical picture of challenge among different phage types of the microbe, signified the variation in their comparative efficacy to control *Salmonella enteriditis* phage type 6A infection. The variation in clinical signs induced among groups inoculated with different inactivated *Salmonella enteriditis* phage types was understandable as there was difference in the virulence of different *Salmonella enteriditis* phage types [10] and hence the immunogenicity.

Depression, anorexia, ruffled feathers, and diarrhoea which were observed in CV0C group indicated that *Salmonella enteriditis* phage type 6A could cause clinical disease in mature SPF chickens. Moreover, it was obvious from clinical signs in inoculated with challenge groups that there was cross protection in the control of the disease. Inactivated *Salmonella enteriditis* phage types 3A and 6A were the most effective in the control of the clinical picture followed by *Salmonella enteriditis* phage types 1, 7, and 35. The commercial vaccines of inactivated *Salmonella enteriditis* had similar potential to mitigate the clinical signs as compared with control challenge groups. However, Huberman et al. [11] reported that there were no clinical signs of depression or disease in chickens vaccinated with (AviPro® *Salmonella* Vac E, ELANCO) (live commercial attenuated *Salmonella enteritidis* vaccine based on strain Sm24/Rif12/Ssq) since vaccination of broiler breeder hens with trivalent inactivated *Salmonella* vaccine had provided a safe and efficacious reduction of both intestinal colonization and organ invasion with related serovars of *Salmonella enterica* following challenge. Subsequently, it could be used for the control programme to reduce contamination rates of poultry flocks and products [12], so inactivated *Salmonella enteriditis* phage types (especially 3A and 6A) could play an effective role in the control of *Salmonella enteriditis* infection in Malaysia. The presence of mortality in the control group CV0C and the absence of mortality in the inoculated and challenged groups gave evidence that inactivated *Salmonella enteriditis* phage types had efficacy to control mortality in specific pathogen-free chickens. There was a decreased of 0.7% and 2.8% in the body weight among chickens of group CV0C in comparison with chickens of group CV0 on day 7 and 14 pc. It indicated that *Salmonella enteriditis* challenge adversely affected the body weight gain.

However, chickens challenged with *Salmonella enteriditis* phage type 6A after inoculation with different inactivated *Salmonella enteriditis* phage types gained better weights than challenged control. It was found that inactivated *Salmonella enteriditis* phage types 3A and 6A were more effective in the control of the adverse action on body weight gain when it was compared to CV0C on days 21 and 28 pi, respectively. However, El-Shall et al. [13] reported that the use of live *Salmonella enteriditis* vaccine had a strong protective effect against *Salmonella enteriditis* challenge especially for growth performance including body weight gain, which supported the suggestion of inactivated *Salmonella enteriditis* phage types 3A and 6A for the control of *Salmonella enteriditis* infection in Malaysia.

One major concern in the strategies for the control of *Salmonella enteriditis* was the colonization of the microbe in different organs such as oviduct, ovaries, liver, spleen, intestine, caecum, and hence, the faecal shedding [12]. The *Salmonella* isolation results indicated a promising efficacy of inactivated *Salmonella enteriditis* phage types. *Salmonella enteriditis* was isolated from 75% of faecal swabs and intestinal contents and 50% of caecum, liver, spleen, and blood samples of the group challenged control CV0C on day 7 pc. The same isolation percentage was observed on day 14 pc apart from only 25% reduction in faecal swabs and liver samples. It indicated the ability of *Salmonella enteriditis* phage type 6A to cause systemic infection even in chickens at the age of 4 weeks old.

All the groups inoculated with different inactivated *Salmonella enteriditis* phage types reduced the organ colonization and faecal shedding. However, inoculation with inactivated *Salmonella enteriditis* phage type 3A and 6A could provide some protection against the challenge and revealed great potential to reduce the bacterial colonization and faecal shedding. Moreover, both *Salmonella enteriditis* phage types 3A and 6A eliminated the bacteria from the liver, spleen, blood, and faecal swabs on day 7 pc. Nevertheless, one caecum and one intestinal content samples were positive in both CV3AC and CV6AC groups on day 7 pc. In addition, on day 7 pc, it was found that the inactivated phage type 3A was able to clear the intestine, whereas *Salmonella enteriditis* phage type 6A cleared the caecum on day 14 pc. Furthermore, the inactivated *Salmonella enteriditis* phage types 1, 7, and 35 showed some potential to prevent the challenge on day 14 pc. However, in a study conducted by Elazomi et al. [14], the protection produced by formalin-killed *Salmonella* harvested from either *in vitro* or *in vivo* conditions when used as vaccine candidate in chickens was found to be statistically insignificant.

It was reported in some previous studies that experimental infection of chickens with *Salmonella enteriditis* could cause intestinal colonization and associated bacterial shedding in faeces for several months [15]. Furthermore, *Salmonella,* with its different serovars, was able to survive and persist in the shed environment [9]. However, in the present study, the inoculation of inactivated *Salmonella enteriditis* phage types 3A and 6A not only reduced the organ colonization but also eliminated the bacteria of challenge in a great number of chickens.

The variation in protection level and other results by different inactivated *Salmonella enteriditis* phage types depended on the factors such as the composition of the adjuvant, the strain of *Salmonella enteriditis*, and the inactivation method. The results of gross lesions and histopathological changes supported the results of the isolation of the microbe. It indicated that *Salmonella enteriditis* was not persistent in the organs. Hence, it was unable to produce gross lesions in different tissues. The absence of lesions in chickens inoculated with challenge of the control might be attributed to the age of chickens at the time of challenge. Further, the number of tissues that showed histopathological changes was higher in control chickens uninoculated and challenged (CV0C) than in chickens inoculated with challenge (CV1C, CV3AC, CV6AC, CV7C, and CV35C). It signified the efficacy of inactivated *Salmonella enteriditis* phage types in the present study.

## 5. Conclusions

It was concluded that different inactivated *Salmonella enteriditis* phage types might partially prevent the chickens against *Salmonella enteriditis* phage type 6A infection. It also provided evidence that inactivated *Salmonella enteriditis* phage types 3A and 6A were safe and efficacious. In addition to that, their inoculation might contribute to high level of protection against *Salmonella enteriditis* phage type 6A infection in chickens.

## Figures and Tables

**Table 1:** Experimental design.

Groups	Inoculation of inactivated SE PTs	Description of inoculated groups	No. of chickens sacrificed on days pi			
			D0	D14	D21	D28
CV1	Inactivated SE PT1	Inoculated and not challenged	—	4	4	4
CV1C	Inactivated SE PT1	Inoculated and challenged	—	—	4	4
CV3A	Inactivated SE PT3A	Inoculated and not challenged	—	4	4	4
CV3AC	Inactivated SE PT3A	Inoculated and challenged	—	—	4	4
CV6A	Inactivated SE PT6A	Inoculated and not challenged	—	4	4	4
CV6AC	Inactivated SE PT6A	Inoculated and challenged	—	—	4	4
CV7	Inactivated SE PT7	Inoculated and not challenged	—	4	4	4
CV7C	Inactivated SE PT7	Inoculated and challenged	—	—	4	4
CV35	Inactivated SE PT35	Inoculated and not challenged	—	4	4	4
CV35C	Inactivated SE PT35	Inoculated and challenged	—	—	4	4
CV0	Control	Inoculated and not challenged	4	4	4	4
CV0C	Control	Noninoculated and challenged	—	—	4	4

SE PT = *Salmonella enteriditis* phage type. Chickens were inoculated at the age of one day old with SE PTs and challenged on day 14 postinoculation.

**Table 2:** Chi-square association for analysis of isolation of *Salmonella* from different tissues of chickens inoculated with inactivated products and challenge on day 7 pc.

	Value	Degree of freedom	Asympototic significance (2-sided)
Pearson's chi-square	21.143	15	0.13
Likelihood ratio	28.103	15	0.92
Number of valid cases	36		

The row of Pearson's Chi-Square explained that value = 21.14 and *p* = 0.13. It meant that there was no statistically significant association between tissue and *Salmonella* isolation on day 7 pc.

**Table 3:** Chi-square association for analysis of isolation of *Salmonella* from different tissues of chickens inoculated with inactivated products and challenge on day 14 pc.

	Value	Asymptotic significance (2-sided)
Pearson's chi-square	19.36	0.20
Likelihood ratio	22.24	0.10
Number of valid cases	36	

The row of Pearson's Chi-square explains that value = 19.36 and *p* = 0.20. It meant that there was no significant association between tissue and isolation of *Salmonella* on day 14 pc.

**Table 4:** Isolation of *Salmonella* in different tissues and the mean for isolation in whole groups of chickens inoculated with inactivated products and challenge on day 7 pc.

Group	Midintestinal content	Caecal content	Cloacal swab	Blood	Liver	Spleen	Mean ± std. deviation
CV0C	0.75	0.50	0.75	0.50	0.50	0.50	0.58 ± 0.13
CV1C	0.50	0.25	0.25	0.00	0.25	0.00	0.21 ± 0.19
C3AC	0.25	0.25	0.00	0.00	0.00	0.00	0.08 ± 0.13
C6AC	0.25	0.25	0.00	0.00	0.00	0.00	0.08 ± 0.13
CV7C	0.25	0.25	0.00	0.00	0.25	0.00	0.13 ± 0.14
C35C	0.25	0.25	0.25	0.00	0.25	0.25	0.21 ± 0.10

Std. stands for standard.

**Table 5:** Means and standard errors for isolation of *Salmonella* in different groups of chickens inoculated with inactivated products and challenge on day 7 pc.

Groups	Mean	Std. error	95% confidence interval	
			Lower bound	Upper bound
CV0C	0.58	0.05	0.45	0.72
CV1C	0.21	0.08	0.01	0.401
CV3AC	0.08	0.05	−0.05	0.22
CV6AC	0.08	0.05	−0.05	0.22
CV7C	0.13	0.06	−0.02	0.27
CV35C	0.21	0.04	0.10	0.32

Std. stands for standard.

**Table 6:** Differences of means for isolation of *Salmonella* between individual groups of chickens, inoculated with inactivated products and challenge, on day 7 pc.

(I) groups	(J) groups	Mean difference (I-J)	Std. error	Sig.	95% confidence interval for difference	
					Lower bound	Upper bound
CV0C	CV1C	0.38∗	0.06	0.02	0.08	0.67
	C3AC	0.50∗	0.07	0.01	0.16	0.84
	C6AC	0.50∗	0.07	0.01	0.16	0.84
	CV7C	0.46∗	0.08	0.03	0.06	0.86
	C35C	0.38∗	0.06	0.02	0.08	0.67
CV1C	CV0C	−0.38∗	0.06	0.02	−0.67	−0.08
	C3AC	0.13	0.06	1.00	−0.17	0.41
	C6AC	0.13	0.06	1.00	−0.17	0.41
	CV7C	0.08	0.05	1.00	−0.19	0.36
	C35C	0.00	0.07	1.00	−0.34	0.34
CV3AC	CV0C	−0.50∗	0.07	0.01	−0.84	−0.16
	CV1C	−0.13	0.06	1.00	−0.42	0.17
	C6AC	0.00	0.00	.	0.00	0.00
	CV7C	−0.04	0.04	1.00	−0.26	0.18
	C35C	−0.13	0.06	1.00	−0.42	0.17
CV6AC	CV0C	−0.50∗	0.07	0.01	−0.84	−0.16
	CV1C	−0.13	0.06	1.00	−0.42	0.17
	C3AC	0.00	0.00	.	0.00	0.00
	CV7C	−0.04	0.04	1.00	−0.26	0.18
	C35C	−0.13	0.06	1.00	−0.42	0.17
CV7C	CV0C	−0.46∗	0.08	0.03	−0.86	−0.06
	CV1C	−0.08	0.05	1.00	−0.36	0.19
	C3AC	0.04	0.04	1.00	−0.18	0.26
	C6AC	0.04	0.04	1.00	−0.18	0.26
	C35C	−0.08	0.05	1.00	−0.36	0.19
CV35C	CV0C	−0.38∗	0.06	0.02	−0.67	−0.08
	CV1C	0.00	0.07	1.00	−0.34	0.34
	C3AC	0.13	0.06	1.00	−0.17	0.42
	C6AC	0.13	0.06	1.00	−0.17	0.42
	CV7C	0.08	0.05	1.00	−0.19	0.36

∗Stands for significance (*p* < 0.05). Sig. stands for significance.

**Table 7:** Isolation of *Salmonella* in different tissues and the mean for isolation in whole groups of chickens inoculated with inactivated products and challenge on day 14 pc.

	Midintestinal content	Caecal content	Cloacal swab	Blood	Liver	Spleen	Mean ± std. deviation
CV0C	0.75	0.5	0.5	0.5	0.25	0.5	0.50 ± 0.16
CV1C	0.5	0.25	0.5	0	0	0	0.21 ± 0.25
C3AC	0.25	0	0	0	0	0	0.04 ± 0.10
C6AC	0	0.25	0	0	0	0	0.04 ± 0.10
CV7C	0.25	0	0	0	0.25	0	0.08 ± 0.13
C35C	0.25	0.25	0	0	0	0	0.08 ± 0.13

Std. stands for standard.

**Table 8:** Means and standard errors for isolation of *Salmonella* in different groups of chickens inoculated with inactivated products and challenge on day 14 pc.

Groups	Mean	Std. error	95% confidence interval	
			Lower bound	Upper bound
CV0C	0.50	0.07	0.33	0.67
CV1C	0.21	0.10	−0.05	0.47
CV3AC	0.04	0.04	−0.07	0.15
CV6AC	0.04	0.04	−0.07	0.15
CV7C	0.08	0.05	−0.05	0.22
CV35C	0.08	0.05	−0.05	0.22

Std. stands for standard.

**Table 9:** Differences of means for isolation of *Salmonella* between individual groups of chickens inoculated with inactivated products and challenge on day 14 pc.

(I) groups	(J) groups	Mean difference (I-J)	Std. error	Sig.	95% confidence interval for difference	
					Lower bound	Upper bound
CV0C	CV1C	0.29	0.08	0.19	−0.11	0.70
	C3AC	0.46∗	0.04	0.00	0.24	0.68
	C6AC	0.46∗	0.08	0.03	0.06	0.86
	CV7C	0.42	0.08	0.06	−0.02	0.85
	C35C	0.42∗	0.05	0.01	0.14	0.69
CV1C	CV0C	−0.29	0.08	0.19	−0.70	0.11
	C3AC	0.17	0.08	1.00	−0.27	0.60
	C6AC	0.17	0.11	1.00	−0.39	0.72
	CV7C	0.13	0.11	1.00	−0.44	0.69
	C35C	0.13	0.09	1.00	−0.32	0.57
CV3AC	CV0C	−0.46∗	0.04	0.00	−0.68	−0.24
	CV1C	−0.17	0.08	1.00	−0.60	0.27
	C6AC	0.00	0.07	1.00	0.34	0.34
	CV7C	−0.04	0.04	1.00	−0.26	0.18
	C35C	−0.04	0.04	1.00	−0.26	0.18
CV6AC	CV0C	−0.46∗	0.08	0.03	−0.86	−0.06
	CV1C	−0.17	0.11	1.00	−0.72	0.39
	C3AC	0.00	0.07	1.00	−0.34	0.34
	CV7C	−0.04	0.08	1.00	−0.45	0.36
	C35C	−0.04	0.04	1.00	−0.26	0.18
CV7C	CV0C	−0.42	0.42	0.06	−0.85	0.02
	CV1C	−0.13	0.13	1.00	−0.69	0.44
	C3AC	0.04	0.04	1.00	−0.18	0.26
	C6AC	0.04	0.04	1.00	−0.36	0.45
	C35C	0.00	0.05	1.00	−0.34	0.34
CV35C	CV0C	−0.42∗	0.06	0.01	−0.69	−0.14
	CV1C	0.13	0.07	1.00	−0.57	0.32
	C3AC	0.04	0.06	1.00	−0.18	0.26
	C6AC	0.04	0.06	1.00	−0.18	0.26
	CV7C	0.00	0.05	1.00	−0.34	0.34

∗Indicated significance (*p* < 0.05). Sig. stands for significance.

**Table 10:** Means and standard deviations for scoring of hetorophilic infiltration and congestion in different tissues of chickens inoculated with challenge on day 7 pc.

Tissue	Mean	Standard deviation	*N*
Ileum	0.10	0.11	6
Caecum	0.13	0.10	6
Bursa of Fabricius	0.17	0.08	6
Liver	0.10	0.11	6
Spleen	0.10	0.11	6

*N* stands for the number of groups (CV0C, CV1C, CV3AC, CV6AC, CV7C, and CV35C).

**Table 11:** Means and standard errors for scoring of hetorophilic infiltration and congestion in different tissues of chickens inoculated with challenge on day 7 pc.

Tissue	Mean	Standard error	95% confidence interval		*N*
			Lower bound	Upper bound	
Ileum	0.10	0.05	−0.02	0.22	6
Caecum	0.13	0.04	0.03	0.24	6
Bursa of Fabricius	0.17	0.03	0.08	0.25	6
Liver	0.10	0.05	−0.02	0.22	6
Spleen	0.10	0.05	−0.02	0.22	6

*N* stands for the number of groups inoculated with challenge.

**Table 12:** Differences of means for scoring of hetorophilic infiltration and congestion induced by *Salmonella* in different tissues of chickens inoculated with challenge on day 7 pc.

(I) tissues	(J) tissues	Mean difference (I−J)	Std. error	Sig.	95% confidence interval for difference	
					Lower bound	Upper bound
Ileum	Caecum	−0.03	0.03	1.00	−0.19	0.13
	Bursa	−0.07	0.04	1.00	−0.27	0.14
	Liver	0.00	0.00	.	0.00	0.00
	Spleen	0.00	0.00	.	0.00	0.00
Caecum	Ileum	0.03	0.03	1.00	−0.13	0.19
	Bursa	−0.03	0.03	1.00	−0.19	0.13
	Liver	0.03	0.03	1.00	−0.13	0.19
	Spleen	0.03	0.03	1.00	−0.13	0.19
Bursa	Ileum	0.07	0.04	1.00	−0.14	0.27
	Caecum	0.03	0.03	1.00	−0.13	0.19
	Liver	0.07	0.04	1.00	−0.14	0.27
	Spleen	0.07	0.04	1.00	−0.14	0.27
Liver	Ileum	0.00	0.00	.	0.00	0.00
	Caecum	−0.03	0.03	1.00	−0.19	0.13
	Bursa	−0.07	0.04	1.00	−0.27	0.14
	Spleen	0.00	0.00	.	0.00	0.00
Spleen	Ileum	0.00	0.00	.	0.00	0.00
	Caecum	−0.03	0.03	1.00	−0.19	0.13
	Bursa	−0.07	0.04	1.00	−0.27	0.14
	Liver	0.00	0.00	.	0.00	0.00

Sig. stands for significance. Std. stands for standard.

**Table 13:** Means and standard deviations for scoring of hetorophilic infiltration and congestion in different tissues of chickens inoculated with challenge on day 14 pc.

Tissue	Mean	Standard deviation	*N*
Ileum	0.20	0.22	6
Caecum	0.23	0.20	6
Bursa of Fabricius	0.33	0.16	6
Liver	0.20	0.22	6
Spleen	0.20	0.22	6

*N* stands for the number of groups (CV0C, CV1C, CV3AC, CV6AC, CV7C, and CV35C).

**Table 14:** Means and standard errors for scoring of hetorophilic infiltration and congestion in different tissues of chickens inoculated with challenge on day 14 pc.

Tissue	Mean	Standard error	95% confidence interval		*N*
			Lower bound	Upper bound	
Ileum	0.20	0.09	−0.03	0.43	6
Caecum	0.23	0.08	0.03	0.44	6
Bursa of Fabricius	0.33	0.07	0.16	0.51	6
Liver	0.20	0.09	−0.03	0.43	6
Spleen	0.20	0.09	−0.03	0.43	6

*N* stands for the number of groups (CV0C, CV1C, CV3AC, CV6AC, CV7C, and CV35C).

**Table 15:** Differences of means for scoring of hetorophilic infiltration and congestion induced by *Salmonella* in different tissues of chickens inoculated with challenge on day 14 pc.

(I) tissues	(J) tissues	Mean difference (I−J)	Std. error	Sig.	95% confidence interval for difference	
					Lower bound	Upper bound
Ileum	Caecum	−0.03	0.08	1.00	−0.42	0.35
	Bursa	−0.13	0.08	1.00	−0.54	0.27
	Liver	0.00	0.00	.	0.00	0.00
	Spleen	0.00	0.00	.	0.00	0.00
Caecum	Ileum	0.03	0.08	1.00	−0.35	0.42
	Bursa	−0.10	0.07	1.00	−0.43	0.27
	Liver	0.03	0.08	1.00	−0.35	0.42
	Spleen	0.03	0.08	1.00	−0.35	0.42
Bursa	Ileum	0.13	0.08	1.00	−0.27	0.54
	Caecum	0.10	0.07	1.00	−0.23	0.43
	Liver	0.13	0.08	1.00	−0.27	0.54
	Spleen	0.13	0.08	1.00	−0.27	0.54
Liver	Ileum	0.00	0.00	.	0.00	0.00
	Caecum	−0.03	0.08	1.00	−0.42	0.35
	Bursa	−0.13	0.08	1.00	−0.54	0.27
	Spleen	0.00	0.00	.	0.00	0.00
Spleen	Ileum	0.00	0.00	.	0.00	0.00
	Caecum	−0.03	0.08	1.00	−0.42	0.35
	Bursa	−0.13	0.08	1.00	−0.54	0.27
	Liver	0.00	0.00	.	0.00	0.00

Std. stands for standard. Sig. stands for significance.

**Table 16:** Comparison of day 7 and day 14 pc with respect to lesions induced by *Salmonella* in all tissues of chickens inoculated with inactivated products and challenge.

	Mean	*N*	Std. deviation	Std. error mean
Day 7 pc	0.19	29	0.36	0.07
Day 14 pc	0.24	29	0.20	0.04

*N* stands for the number of tissues. Std. stands for standard.

**Table 17:** Paired-samples *t*-test for the comparison of day 7 and day 14 pc with respect to lesions induced by *Salmonella* in all tissues of chickens inoculated with inactivated products and challenge.

	Paired differences					*t*	df	Sig. (2-tailed)
	Mean	Std. deviation	Std. error mean	95% confidence interval of the difference				
				Lower	Upper			
Day 7 pc – day 14 pc	−0.06	0.33	0.06	−.018	0.07	−0.89	28	0.38

df stands for degrees of freedom. *t* stands for *t*-value. Sig. stands for significance level.

**Table 18:** Antibody by time (ELISA units) of chickens from different inactivated *Salmonella enteriditis* phage types' groups throughout the experiment.

Groups	Mean titer	GMT	CV%	Mean titer	GMT	CV%	Mean titer	GMT	CV%
	2 weeks			3 weeks			4 weeks		
CV0	2	2	85	14	11	85	8	6	78
CV1	2	2	150	17	13	64	14	7	94
CV3A	11	4	158	67	35	143	17	10	114
CV6A	12	8	59	24	24	28	15	8	103
CV7	16	15	53	10	4	113	10	6	99
CV35	14	13	51	28	27	28	22	17	76
CV0C	—	—	—	165	103	68	16	14	64
CV1C	—	—	—	28	27	35	38	19	71
CV3AC	—	—	—	18	16	48	1359	380	108
CV6AC	—	—	—	21	17	89	44	43	133
CV7C	—	—	—	10	5	109	18	14	78
CV35C	—	—	—	8	7	63	85	50	110

## Data Availability

The data used to support the findings of this study are included within the article.
